# A new approach to gathering pharmaceutical market data to support policy implementation and access to medicines: as demonstrated by malaria medicines in Zambia

**DOI:** 10.1186/s12936-018-2594-9

**Published:** 2018-11-29

**Authors:** Renia Coghlan, Peter Stephens, Bernice Mwale, Makomani Siyanga

**Affiliations:** 1 TESS Development Advisors, Geneva, Switzerland; 2 IQVIA, London, UK; 3 Zambia Medicines Regulatory Agenda, Lusaka, Zambia

**Keywords:** Medicines, Pharmaceuticals, Market dynamics, Market structure, Pharmaceutical market data, Private sector, Malaria, Zambia, Drug utilisation research

## Abstract

**Background:**

The steady supply of quality, affordable medicines is a pillar of a functioning health system. In addition to the public sector, the private, mission and not-for-profit sectors often serve a large part of the population in Africa. However, while there is generally systematic recording of public sector supply of medicines, detailed, systematic and reliable national market data including these non-public sectors are not commonly available in most countries in Africa. Understanding the total market is a missing part of the access puzzle: without this information, policy makers and health practitioners are not able to fully measure the impact of interventions, measure access to effective products, or fully evaluate the rational use of medicines. This article reports on a unique innovation which provides routine, national-level data on the total pharmaceuticals market, through a system which can be replicated elsewhere. It demonstrates how national-level market data contribute to the evidence base for policies on access to essential medicines, using the Zambian anti-malarial medicines market as a case study.

**Methods:**

A new, routine national database on pharmaceutical market size and structure was established through a multi-partner collaboration. Information was extracted from import authorizations and allows for information on local manufacture. Data included value and volume of products as well as pack details, manufacturer and importer. The system was continually updated: data for this analysis were extracted for 6 years: 2009–2014 inclusive. Data were analysed using Microsoft Excel and validated against other sources including donor procurement data. Analysis included public and private sector markets. The policy relevance was demonstrated through analysis of four aspects of national policies on access and rational use of malaria medicines: (i) volume of product relative to disease burden; (ii) distribution by sector relative to treatment-seeking; (iii) consistency of products with respect to national policy guidelines; (iv) market concentration as a proxy for security of supply.

**Results:**

The system developed provides the first accurate, systematic data on the breakdown of a national pharmaceutical market in an African context. The total value of the anti-malarials market in Zambia, including all sectors, was USD 5.5–6 million. This included 22 different molecules or combinations, produced by 56 different manufacturers, with 142 different permutations of molecule/manufacturer/strength. Such data provide a complementary mechanism to confirm key trends in malaria treatment and control in Zambia: (i) sufficient supply relative to disease burden, (ii) value and volume of the private/non-profit sector; 29%–2% of market value and 17%–2% of market volume (from 2009 to 2014), (iii) dominance of the 3 molecules recommended in the national treatment guidelines; and (iv) an evidence-base for national discussions on medicines quality, security of supply and rationale use. The system extracts information on all medicines and therefore could be used to analyse other therapeutic classes. Data have been used for several policy purposes, notably by ZAMRA to monitor the quality of products in Zambia, monitoring implementation of WHO Resolutions on artemisinin monotherapy as well as monitoring trends in product choice across sectors.

**Conclusion:**

Routine data are important for researchers and policy makers alike. This study shows how medicines data can be systematically gathered at national level—comprising range, volume and value in the public, private and not-for-profit sectors—to monitor more detailed trends in the market and allows triangulation of supply-side data against other sources. This systematic approach can contribute significantly to support access to medicines, monitor treatment and public health policies and create healthy markets. It can be used to monitor changes between therapeutic areas, for example the impact of improved malaria treatment on the use of antibiotics in the context of anti-microbial resistance monitoring. As data contain commercially confidential information, appropriate safeguards should be put in place to balance public health and commercial interests.

## Background

Malaria is a high burden disease in many countries of Africa. It is important that it is treated quickly to achieve good clinical outcomes, and that it is treated correctly to prevent the development of drug resistance. As a disease with a major impact on the poor, it is essential that appropriate, effective treatments are affordable. Malaria treatment should be systematically available in the public sector in endemic countries, and is often also sought in the private sector. There is typically a paucity of routine pharmaceutical data relating to the private sector in low-income countries, making it more difficult for policy makers to easily measure the types, volumes and affordability of medicines used in that sector, and to target appropriate interventions around medicines’ quality, access, rational drug use and affordability.

This article presents a new system which addresses a missing part of the access puzzle: it reports on a unique innovation which provides regular, national-level data on the total pharmaceuticals market in an African country (Zambia), through a system which can and has been replicated elsewhere. This work explores a subset of the data collected, examining the malaria medicines market in Zambia as a case study of how total market data completes the evidence base required for policy making. The database contains all pharmaceuticals and similar analyses could therefore be applied to other therapeutic areas or classes, including antibiotics.

The World Health Organization (WHO) defines universal health coverage (UHC) as ‘*ensuring that all people have access to needed promotive, preventive, curative and rehabilitative health services, of sufficient quality to be effective, while also ensuring that people do not suffer financial hardship when paying for these services*’ [1].

Key among the tools available to healthcare workers to support preventive and curative interventions are medicines, notably essential medicines, i.e. those which satisfy the priority healthcare needs of the population. The WHO references essential medicines as follows: ‘*essential medicines are intended to be available within the context of functioning health systems at all times in adequate amounts, in the appropriate dosage forms, with assured quality, and at a price the individual and the community can afford*’ [2]. Based on a global model essential medicines list [3] developed by the WHO, each country develops its own list taking into account national requirements and priorities. Among the products on the WHO model list and many African countries’ lists are anti-malarial medicines: critical in the fight against this disease.

Access to medicines is a core concept within universal health coverage and is defined as ‘*medicines (being) continuously available and affordable at public or private health facilities or medicine outlets that are within one hour’s walk of the population*’ [4]. An important component of this definition is the inclusion of private health facilities or other medicine outlets. Indeed, a large proportion of the population may initially seek treatment outside the public sector [5–7]. The importance of this sector varies between countries and across interventions. The Malaria Indicator Surveys (MIS) [8] provide a useful common assessment of treatment-seeking in the public or private sectors for children with fever under the age of 5. Instances range from 3.5% of children for whom treatment was sought in the private sector in Burkina Faso [9], to around 70% of children under age 5 in Nigeria taken to a non-public sector source when seeking treatment for fever [10].

Malaria is endemic to large parts of Africa and has long represented one of the leading causes of morbidity and mortality in the region. As such, malaria was recognized as a specific development concern in the millennium development goals [11], which called for a concerted effort to reduce the burden of disease by 2015 (MDG target 6c), and subsequently the Sustainable Development Goals (SDG goal 3) [12]. There were an estimated 212 million malaria cases worldwide in 2015 [13], with the bulk of the disease burden—90%—in Africa. This burden is slowly falling: malaria incidence rates (new malaria cases) have decreased by 21% globally between 2010 and 2015 [13], indicating that the right interventions can achieve impact. Note: the year 2015 is taken as a reference as the first year after the data analysed in this paper, 2009–2014. Malaria is particularly dangerous for children under 5 years of age and pregnant women. As a disease which can kill quickly if left untreated, there has long been a tradition of presumptive diagnosis of malaria in young children with fever.

The WHO has led global efforts to address and reduce this burden of disease through the development and country-level dissemination of new policies and treatment guidelines. Among these, special reference is made to the 2006 and 2010 WHO Guidelines for the Treatment of Malaria (STG) [14, 15] which first recommended the use of artemisinin-based combination therapy (ACT) for the treatment of malaria in 2006, then in 2010 a recommendation to confirm diagnosis using microscopy or rapid diagnostic tests (RDT). Reference is made to the WHO 2010 guidelines for malaria rather than the 2015 version, as the data analysed covered a period prior to 2015, i.e. falling within the earlier guidelines. The development of new treatment and prevention options, diagnostic tools and policies has significantly changed the range of options available to countries to tackle this disease. On average, based on data from DHS surveys from 18 malaria-endemic countries and carried out between 2013 and 2015, as stated in the World Malaria Report 2015 (page xii), 47% of children with malaria now receive ACT [16].

Zambia has been in the forefront of addressing malaria as a key health challenge. It has invested heavily in malaria control activities and updated its treatment guidelines accordingly. The national treatment guidelines produced by the Zambian Ministry of Health (MoH) clearly indicate the recommended treatments which should be used for the treatment of uncomplicated malaria and severe malaria [17]. These state that artemether–lumefantrine (AL) should be used as first-line treatment for uncomplicated malaria, or sulfadoxine–pyrimethamine (SP) for children under 5 kg of weight and pregnant women. Dihydroartemisinin–piperaquine (DHA–PQP) is identified as an alternative first-line. Oral quinine should be given in case of failure of the first-line treatment for uncomplicated malaria. Injectable artesunate (IV Inj. AS) is the first-line recommended treatment for severe malaria, with intramuscular (IM) artemether or IV or IM Quinine suggested as alternatives if IV Inj. AS is not available. The 2014 change to the treatment guideline added the new alternative first line for uncomplicated malaria and a change from quinine to Inj. AS or IM artemether for severe malaria as the preferred first-line for severe malaria. A key question from a supply-side perspective is ‘*do the products available on the market comply with the national treatment guidelines and national policies?’*. This is important to ensure that the treatment available to all, irrespective of sector, is effective and affordable.

The Zambia Medicines Regulatory Agency (ZAMRA) is responsible for licensing medicines for use in Zambia and monitoring their use, e.g. through post-marketing surveillance. The list of products approved for use in Zambia may be found on the national medicines register produced by ZAMRA every year [18]. In addition, ZAMRA has responsibility for approving all import requests for medicines through the medicines inspection process. Requests for approval should be submitted to, and authorized by, ZAMRA before a consignment of imported medicines is released onto the market. ZAMRA also has responsibility for inspecting manufacturers and importers to ensure their compliance with local regulations (note—Zambia has a very small pharmaceutical manufacturing base; over 99% of volume and value of products are imported). Finally, it also has a public health remit to ensure the quality and general availability of medicines within Zambia, in conjunction with the Ministry of Health. ZAMRA has regular contact with a range of stakeholders, including manufacturers and importers, and therefore requires a wide evidence base in order to engage in constructive dialogue and to monitor the impact of change which might negatively affect the continuous availability of medicines in the country.

The evidence base required by both the Ministry of Health policy makers and ZAMRA should ideally be provided through a core set of routine data. However, malaria has long been a complicated disease to track and measure, in part due to a tradition of presumptive (rather than confirmed) treatment. Without good diagnosis and routine data, health practitioners faced difficulties in measuring disease burden and changes in disease patterns. There have been a number of important investments to improve systematic data. The MIS surveys [8] provide a baseline and regular update on information about parasitaemia rates, treatment-seeking and treatment provision. As the private sector has long been recognized as a key player in service provision [5–7], the ACT Watch surveys [19] were established to provide some insights into market dynamics for several countries in Africa including Zambia. Such data provide an indication of supply and price dynamics at outlet level. However, there was still a core gap in understanding national level supply dynamics, including the balance of supply between the public and private sectors.

The examples provided in this manuscript highlight how national market data can provide an important source of evidence to support, question or confirm investments to ensure access to essential medicines and support continued drug utilization research [20].

## Methods

A new, routine national database on pharmaceutical market size and structure was established through a collaboration between the Zambia Medicines Regulatory Agency and IMS Health (now IQVIA), initiated by Medicines for Malaria Venture (MMV), and with the involvement of TESS Development Advisors in the design of the system, methodology, country outreach and data analysis. The collaboration was established in 2008 and involves these four parties working together to provide systematic and detailed information on the pharmaceutical market in Zambia. This paper is based on a subset of data from the system.

Information on pharmaceutical imports was gathered from the national regulatory authority—ZAMRA—as well as from the central medical stores (Medical Stores Ltd, MSL). The information combines data extracted from approved applications by importers with direct deliveries into the central medical stores. It includes information from the public sector, the private sector, not-for-profit health providers as well as imports by individual hospitals. The data collected cover all pharmaceuticals. As indicated above, Zambia has very little local manufacturing of pharmaceuticals, notably none of major relevance for malaria in the time-period under review. However, the system has also been explored in African countries with larger local manufacturing sectors and can be easily adapted to include local manufacture data.

Data are available on a number of key fields essential for market analysis and drug utilization studies, including molecule (INN or generic name), manufacturer, brand name, strength, formulation, pack size, volume and value. Value is converted from the invoice currency to USD using the latest month’s exchange rate and applied across the whole time-period: prices are thus presented using a constant USD value across the dataset.

The data are entered into a specialized software by ZAMRA staff. The data are then cleaned and standardized by IMS Health (IQVIA). Quality control is carried out by IMS Health (IQVIA), TESS Development Advisors and ZAMRA. The database is updated quarterly. Access to the data is available to the Zambian Authorities and IMS Health (IQVIA), and measures are included to protect commercial confidentiality. The principles of the system can be adapted to non-commercial situations and suppliers as long as commercial confidentiality is protected.

For the purposes of this study, data on the anti-malarials imported to Zambia were extracted from the full dataset into Microsoft Excel software and analysed for trends over the period 2009–2014 inclusive. This period offers an interesting insight into Zambia’s initiatives to control its malaria burden, and move towards elimination in selected parts of the country. Full, detailed data are available in the system for the entire calendar year from January 2009 and thus overlap with the period of policy change in Zambia.

As the data relate to availability of product, i.e. on the supply side of the market, the analysis focuses on the relation between these supply-side data and the following four questions: (i) volume of product relative to disease burden; (ii) distribution by sector relative to treatment-seeking; (iii) consistency of products with respect to national policies; (iv) market concentration as a proxy for security of supply. Where possible, results were compared against demand-side or epidemiological data to assess how routine supply-side data could complement or triangulate other data sources.

## Results

### Overview of the market

The value of the market for anti-malarial medicines in Zambia ranged from a low of just over USD 2 million in 2011 to a high of USD 15 million in 2014. The other 4 years (2009, 2010, 2012, 2013) remained stable between USD 5.5 million–6 million. This reflects donor funding-related dynamics, discussed further below. The market comprised 22 different molecules (or combination molecules), of which 19 are indicated for the treatment of malaria and 3 used for prophylaxis, i.e. preventive, purposes. At least 56 different manufacturers and 28 different importers are involved in the supply chain for anti-malarials in Zambia in some way.

### Market size relative to disease burden

A key question in an access discussion is ‘*is there enough product going in at the top of the supply chain?*’; insufficient product at the top of the chain will lead to later supply interruptions at lower levels, higher prices or other challenges. The total amount of product for the treatment of uncomplicated malaria was therefore analysed relative to the number of reported presumed and confirmed cases of uncomplicated malaria (see Table 1) [13]. In all years, the supply of products was easily sufficient to meet, and in fact exceeds reported medical need.

**Table 1:** Ratio of treatments for uncomplicated malaria compared to disease burden

	2009	2010	2011	2012	2013
Reported cases (presumed and confirmed, uncomplicated malaria)	2,976,395	4,229,839	4,607,908	4,695,400	5,465,122
Imports of all treatments (to the nearest 100,000	6,500,000	5,400,000	6,500,000	11,00,000	7,800,000
Ratio all treatments/cases	2.19	1.27	1.40	2.34	1.44
Imports 1st line treatment	6,198,899	5,127,519	6,296,924	10,820,671	7,647,384
Ratio 1st line treatments/cases	2.08	1.21	1.37	2.30	1.40

### Market data by sector as compared to treatment-seeking behaviour

There is a very strong consensus among public health and malaria experts in Zambia and elsewhere that malaria is generally treated in the public sector in Zambia, supported from information from national surveys such as DHS and MIS. The authors sought to explore how far the market—supply side—data confirm this widely-held understanding. A key challenge in many African countries is that there is no single, easy to analyse source of total market data to measure availability of product in different sectors. This system provides the authorities in Zambia with easy access to such information in a measurable way for the first time.

The market for anti-malarials in Zambia may be divided broadly into two major sectors: products brought in through the central medical store, i.e. MSL, and products brought in mainly through the private sector. Each sector can then be divided into further subsectors, notably government hospitals versus faith-based facilities for MSL imports and larger importers versus more ad-hoc importers in the private sector. Table 2 provides further details on market share for the public sector as compared to the private sector. The public sector did indeed account for the large majority of all imports; up to 98% of value and volume of anti-malarials in 2014.

**Table 2:** Market share by sector (public versus private)

Market share	2009	2010	2011	2012	2013	2014
USD value (%)						
Public sector imports (MSL)	90	71	81	90	98	98
Private sector imports	10	29	19	10	2	2
Total units (%)						
Public sector imports (MSL)	85	96	75	88	73	76
Private sector imports	5	4	25	12	27	24
Total standard units (%)						
Public sector imports (MSL)	83	93	96	95	98	98
Private sector imports	17	7	4	5	2	2

These data were compared to a reliable source of information on treatment-seeking behaviour in Zambia, using the Malaria Indicator Surveys (MIS). The MIS confirms that 82% of product distributed to children under 5 were provided at a government facility or community health workers (served by the government supply chain) in 2010 as compared to 13,8% in a private facility [21]. Results from the 2012 MIS survey were very similar with 82.6% of drugs provided to children under 5 came from government-supplied sources while only 7,6% came from the private sector [22]. Supply-side data indicate that the private sector comprised 29% of market value and 4% of units sold in 2010, and 10% market value or 12% of total units in 2012. It should be noted that the value of products supplied through each sector differs depending on the breakdown of product mix as the private sector supplies more high value products, including prophylaxis products, which pushes up the relative share of market value.

### Alignment of market with national policies

A key question from a public health perspective (as opposed to a market or commercial perspective) is ‘*how closely does the market follow national medicines and health policies?*’. For the purposes of this paper, these policies were divided into three categories: marketing authorizations (which regulate the right to sell a medicinal product); the essential medicines list, which focuses on core products to address major health priorities; and national treatment guidelines, produced by the national health authority, in the form of the Ministry of Health, to guide choice of product. These three examples represent key aspects of medicines management: regulatory, supply and clinical guidelines. Despite the confirmed dominance, in this example, of the public sector—which ought to follow national policies—it is important to explore how the market as a whole aligns with such policies, as this impacts the choices and interventions by the national authorities to address any variations or gaps.

Medicines must be registered with ZAMRA, as required by Medicines and Allied Substances Act (No. 3) of 2013 modified by the SI 57 of 2017, before they may be distributed in the country either in the public or private sectors. A product can be registered in Zambia under the same registration number for pack sizes. For example, ZAMRA registers a box of AL 6 tablets and 12 tablets from the same manufacturer and the same strength under the same number. However, each product should be differentiated by molecule, strength, dosage form and manufacturer.

The study, therefore, examined the coherence between the national medicines register and imports in order to assess how closely this requirement is met in Zambia. It should be noted that the register available at the time of analysis was the 2015 register. It is possible that some of the medicines which were not identified were on the register in earlier years but withdrawn in 2015: feedback on this was sought from the ZAMRA registration team, which indicated that no malaria medicines were changed from the 2014 register. A total of 142 individual manufacturer/molecule/strength combinations were identified—i.e. products which should be registered with ZAMRA. 18 products were listed as having no manufacturer name identified. As MSL does not identify manufacturer, and these were MSL-related products, it was assumed that these were already accounted for among the 142 combinations. If a product was identified by manufacturer and molecule but no strength was stated, it was assumed that it was of the same strength as the same product supplied by the same manufacturer on a different occasion.

A total of 42 products were identified for which the manufacturer was not found on the register. In most cases, this is believed to be a coding error as the manufacturer stated in such cases was also indicated elsewhere as an importer. 64 products, or 45% of products, were fully identified on the register, i.e. by manufacturer, molecule and strength. Allowing for corrections of the possible coding errors which can be cleaned in the system as well as gaps due to data not captured by MSL, 87% of all anti-malarials imported could be identified in the 2015 register. It was also noted that some importers of donor-funded or MoH products systematically do not submit import requests on the (incorrect) belief or tradition that they may be exempt. This issue has been noted by the Ministry of Health and ZAMRA to be clarified with the relevant agencies, as per the medicines registration (importation and exportation) legislation SI 57 of 2017.

Another highly relevant policy document is the Essential Medicines List (EML), which provides an indication of those products which are considered essential by the Ministry of Health of Zambia and thus should be available at different levels of the health system. The 2012 version of the Zambian EML identifies the following products for the treatment of malaria: AL, SP, quinine tablet and quinine injection. All the anti-malarials identified in the EML were brought into Zambia each year with the exception of quinine injection in 2009. Further examination of the data with the MoH and MSL indicated that a large supply of quinine injection for treatment of severe malaria had been imported in late 2008 and, therefore, no further supplies were imported in 2009 to avoid overstocking and product expiry.

The treatment guidelines for malaria are very clearly defined by the National Malaria Elimination Center (NMEC) of Zambia. These are described in the introduction and broadly center around three main products in the period 2009–2014: artemether–lumefantrine tablets (AL) for treatment of uncomplicated malaria; sulfadoxine–pyrimethamine tablets (SP) for preventive treatment in pregnancy (IPTp); injectable quinine for treatment of severe malaria. NB: malaria treatment guidelines were updated in 2015 by WHO (globally) and NMEC (for Zambia): these were not considered these as they fall outwith the period of the dataset examined.

**Fig. 1:**
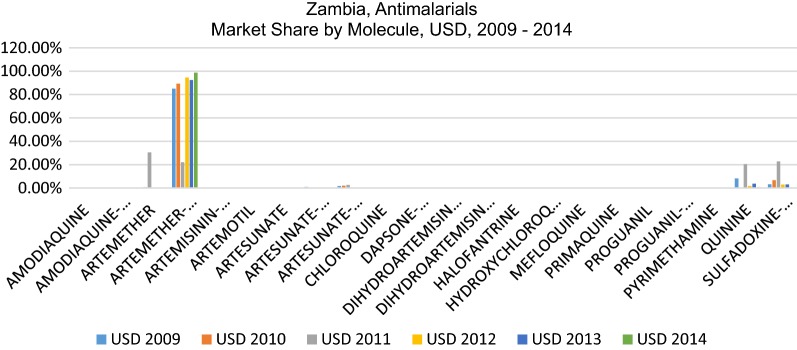
Percent market share by molecule, USD value, 2009–2014

**Fig. 2:**
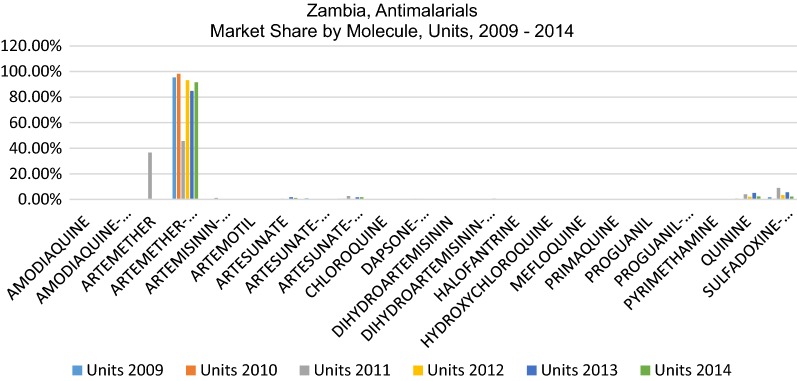
Percent market share by molecule, units, 2009–2014

**Fig. 3:**
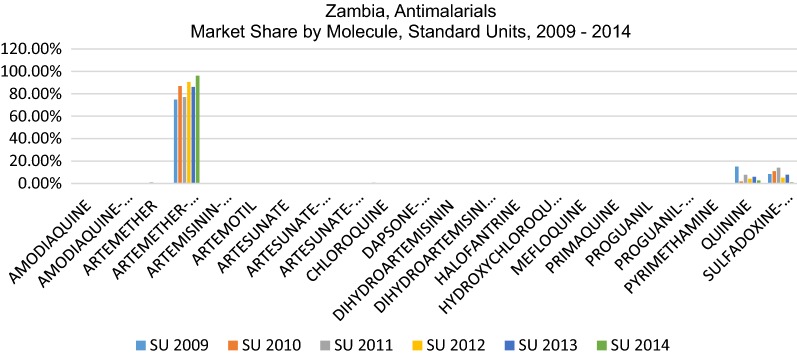
Percent market share by molecule, standard units, 2009–2014

Despite the 22 different molecules/combinations which comprise the total market, only 3 molecules have any significant market share. This pattern is replicated whether market share is calculated based on value (USD), units (packs), or standard units, a unit defined by IMS Health (IQVIA) to represent the smallest normal unit of consumption, e.g. one tablet, one vial/ampoule or 5 ml of liquid. As each measure is used for different purposes, all three are provided in this analysis. Figures 1, 2 and 3 show the very significant dominance of the three key products on the national treatment guidelines (AL, SP and quinine).

**Fig. 4:**
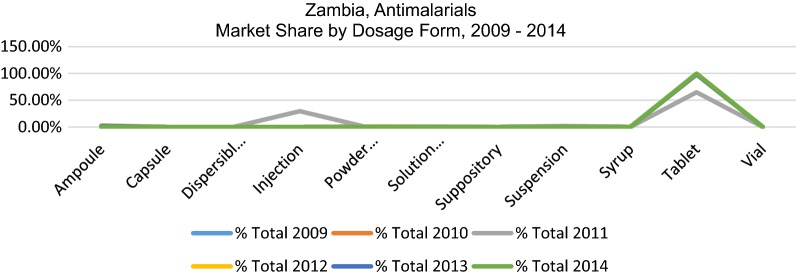
Percent market share by dosage, units, 2009–2014

The system developed allows for analysis of the data by detailed market segment, for example by molecule, form, strength and value or volume market share as well as by sector (public, private and non-profit). This allows analysis of the above-mentioned trends in more detail. The market for anti-malarials in Zambia is very largely dominated by tablet form, which represent between 97.9 and 99.6% of the total market for the years analysed with the exception of 2011 where there was a significant shift to injectables, as shown in Fig. 4. This spike in injectables is explained by the decrease in availability of the first-line, and an increase in treatment using products normally reserved for severe malaria.

Breaking the data down further, it is clear that the market is heavily dominated by one combination: AL tablets at the strength 20/120 mg, as shown in Table 3. Again, it is noted that the treatment guidelines specifically recommend the tablet form for AL and SP, indicating that the market closely follows the recommended treatment guidelines.

**Table 3:** Market share of AL tablets 20/120 mg

AL tablets, 20/120 g; market share	2009	2010	2011	2012	2013	2014
Percent total USD: value (%)	84	88	20	94	92	98
Percent total: units (%)	95	97	43	92	81	86
Percent total: standard units (%)	75	86	77	91	86	96

**Fig. 5:**
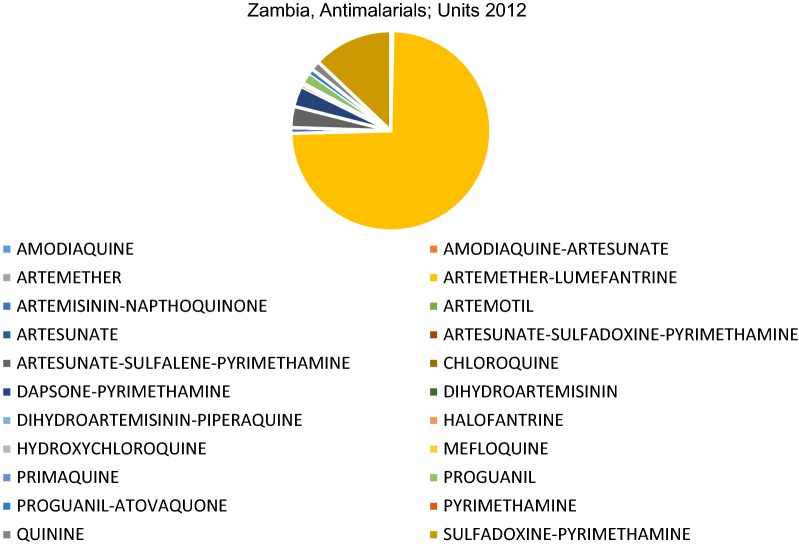
Private sector, percent market share by molecule, units, 2012

As seen above, the public sector is largely dominated by AL as the first-line treatment (up to 99% of total volume at MSL in 2014). The private sector carries a wider range of products, including 22 different molecules. However, AL still dominates even in the private sector: data for market split for 2012 in Fig. 5 give an indication of the dominance of this product.

Health and regulatory authorities will also be interested in changes in the market, notably with respect to less effective products: the private sector is marked by a decrease in the supply of chloroquine (CQ), SP, Artesunate-SP and Artemether from 2009 to 2014, towards a greater demand for AL. CQ had a 3% market share in 2009 dropping to zero in subsequent years, while SP dropped from 21% of market volume in 2009 to 1.5% in 2014. Such information is essential to the regulatory authority and Ministry of Health to monitor the rationale use of medicines.

Finally, information with respect to a specific public health priority for WHO was analysed, namely the withdrawal from the market of artemisinin monotherapy as requested in 2007 World Health Assembly Resolution 60.18 [23]. The level of detail available in the dataset clearly indicates that no oral artemisinin monotherapy was legally imported into Zambia after July 2009.

### Market concentration as a proxy for security of supply

In view of the importance of essential medicines for public health goals, security of supply was measured by use of a proxy: the diversity of manufacturers providing these essential medicine products to Zambia. The results are shown in Table 4. The 22 molecules identified on the Zambian market are produced by at least 56 different manufacturers. A full analysis by manufacturer is difficult, as MSL does not routinely record manufacturer: such data are available separately through other sources such as MOH information. Among the 56 identified manufacturers, only 12 to 18 each had a market share of above 1% in the private sector on an annual basis, and 5–6 manufacturers were responsible for 75% of the products supplied to the private sector each year.

**Table 4:** Number of manufacturers by product type recommended on EML

Molecule	Form	Strength	H/S level	Category	Nr manufacturers supplying product
Artemether–lumefantrine	Tablet	20 mg + 120 mg	I–IV	Vital	7 manufacturers (4 with WHO PQ)
Sulfadoxine–pyrimethamine	Tablet	25 mg + 500 mg	I–IV	Vital	9 manufacturers
Quinine	Tablet	300 mg	I–IV	Vital	8 manufacturers
Quinine	Injection	300 mg/1 ml (2 ml)	I–IV	Vital	3 manufacturers

There are 28 different importers involved in supplying the public and mission sectors through MSL and 50 importers supplying the private sector, other not-for-profit organizations or specific (or individual) clients. However, detailed analysis of the data indicates that there is a much greater concentration in the market than this headline number of importers would suggest. Indeed, among these 50, only 9 importers showed an individual market share worth over 1% of total value for all 6 years. This implies that there were a number of importers who either only sporadically import anti-malarials or who bring in negligible quantities each year. Table 5 shows the market concentration by importer each year.

**Table 5:** Concentration of market among importers 2009–2014

	2009	2010	2011	2012	2013	2014
Market concentration: by USD value						
Number of importers each bringing in at least 0.1% of total market	32	30	28	22	25	18
Number of importers collectively responsible for over 80% of total imports	2	4	7	3	3	2
Market concentration: percent of total units						
Number of importers each bringing in at least 0.1% of total market	23	25	29	29	26	24
Number of importers collectively responsible for over 80% of total imports	1	3	6	2	7	4
Market concentration: percent of total standard units						
Number of importers each bringing in at least 0.1% of total market	27	29	26	26	21	20
Number of importers collectively responsible for over 80% of total imports	3	3	3	3	3	2

## Discussion

These data provide, for the first time in a sub-Saharan African context, a full overview of the pharmaceuticals market at national level. Understanding the supply of medicines is essential in public health. Such detailed data offer new evidence to explore access drivers and bottlenecks. Data of this kind can support drug utilization not only by providing information for comparisons of medicines supply versus case incidence over time, but more importantly provides an overview of what is happening across the entire market, thus giving an insight into both total volumes of medicines available for a certain indication (e.g. for uncomplicated malaria) as well as the specific classes of drugs used, notably the split between newer, more effective products and potentially less effective molecules.

The selection of malaria data in Zambia provides a very simple case study, showing how even in the case of what is apparently a clear-cut dynamic—“malaria is treated in the public sector”—additional information at a detailed level can help guide policy makers.

The data show that the market does supply sufficient product to treat all suspected and unconfirmed cases in the country. These numbers, provided as routine data, can be used for a number of different purposes including supply monitoring, donor reporting and framing behaviour-change interventions to encourage rationale drug use. What stands out from these numbers is the differential between the number of cases reported and the number of treatments supplied (mainly through the public sector at the request of the Ministry of Health). This differential highlights several dynamics in malaria treatment: first, the move from presumptive to confirmed diagnosis, which was rolled out in 2009–2010 nationwide in Zambia. The system has since between adapted to capture information on supply of certain diagnostics, which would also help measure how many diagnostic tests were used relative to suspected cases, as well as the number of treatments supplied. Second, the data may draw attention once again to issues around routine case reporting. This is important not only in the malaria field, but also in other areas, such as tuberculosis (notably paediatric TB) or the use of antibiotics for example. Finally, routine data of this nature, provided on a quarterly basis, allows more detailed tracking of total volumes which can contribute to better planning to avoid over- or under-stocking, stock-outs or expiry.

The particular relevance in this case is not only the number per se, but also the system-based approach. In the case of Zambia, where malaria treatment is widely accessed in the public sector, one could assume that using MSL, or public supplies data, would be a good indicator for sufficiency of supply. However, by providing data which combine all sources of produce, the system may also be used to assess other situations: diseases including non-communicable diseases such as diabetes in Zambia which is treated in both the public and private sectors. The system has also been implemented in Uganda, where the private sector plays a greater role in treatment provision and 57% of children are taken to a private provider to access treatment for suspected malaria. In such cases, it is no longer possible to rely entirely on public sector data: a total market approach, therefore, provides the insight required to ensure that there is enough product flowing through the system irrespective of choice of service providers.

The initial wide-lens view of general uses, zooming into malaria-specific data for Zambia also show how such a system of this type can drill down to very specific information which may affect only a few categories of products. Following the switch to artemisinin-based combination therapy in 2005, funding for anti-malarials in the public sector has been heavily dependent on international donors notably the Global Fund (GF) and the US President’s Malaria Initiative (PMI). This has influenced the product selection and product supply, as these donors require that products should meet strict criteria. In addition to being on the treatment guidelines (unless exceptions are requested), the products must meet quality criteria, for example approval by WHO Pre-Qualification or from a Stringent Regulatory Authority (SRA). These data can support reporting requirements to access international donor funds.

The impact of this external funding is particularly noticeable in 2011 and 2014. Zambia was subject to a temporary suspension of its Global Fund grant for malaria in 2010, thus significantly impacting the volume and value of product procured in 2011 although PMI stepped into cover some of the shortfall. The data show an important drop in the volume of AL tablets, the first line product procured by MSL using Global Fund monies in direct relation to the suspension of the Global Fund grant. This gap is partially filled by supplies of artemether, also an artemisinin-based product, used to treat severe malaria, and SP which was not the first-line treatment but used to treat uncomplicated malaria. PMI also stepped into cover some AL shortfall. The downturn of 2011 is compensated in 2014 reflecting the time for funding from donors to flow again and for the supply pipeline to be refilled, thus creating an unusual bubble of increased supply for that year. Easy access to routine data of this kind can reduce the time that civil servants spend on gathering information for reporting purposes.

The data also allow national authorities to monitor and explore issues around policy compliance in greater detail and with a more robust evidence base on which to engage discussions with national stakeholders. The data clearly indicate that in the case of regulation where the system highlights a small number of gaps in ZAMRA’s systems. During the period under review a new pharmaceuticals law was passed and ZAMRA has been upgrading its systems and internal operating procedures: it has recognized the need to improve on the old software systems which form the base of the regulatory database. The system has helped ZAMRA staff identify specific areas for upgrade, and provided the evidence required to build a business case for further investment, resource allocation, training and upgrading. While flagging internal issues, the system also allows ZAMRA to identify and address gaps in compliance with national guidelines which may be caused inadvertently by other stakeholders. For example, certain importers of donor-funded goods do not systematically register the import requests with ZAMRA. This may happen as a result of implementation of older practices which allow products to be traced in other ways (e.g. through MSL data), but do not fully comply with existing protocols and legislation [see the legislation in force: the Medicines and Allied Substances Act (Importation and Exportation) Regulations, 2017, SI 57 of 2017]. Identifying and evaluating clear examples of this kind allows both parties (ZAMRA and the importer) to address this in a constructive manner.

ZAMRA recognizes a number of other immediate benefits to these data, including the monitoring of small imports; post-marketing surveillance of products to ensure that the products received import approval; efficient allocation of ZAMRA resources; sharing information with other regulatory authorities. Having access to detailed information on import requests which can be accessed and analysed in real time allows ZAMRA to follow up on queries from multiple stakeholders in the health field, whether from the Ministry of Health to check on compliance with WHO recommendations, to the annual meeting with importers, to queries from potential investors on market growth. Having trend data allows ZAMRA to confirm assumptions about the importance of certain stakeholders. For example, knowing that only 9 importers represent over 1% of market share each allows ZAMRA to follow up closely on communications to the industry, while also ensuring that ad-hoc importers of key medicines are fully informed of and aligned with current requirements.

With respect to the essential medicines list and national treatment guidelines, the data clearly indicate that the public sector, unsurprisingly, is following national guidelines. More importantly, it was noticeable that CQ, which is considered ineffective in the treatment of malaria, has entirely lost market share in the private sector and SP is no longer a drug of choice. The ability to monitor trends in product choices provides important insights to monitor the impact of public health campaigns and assess where further interventions maybe required. For example, the move away from CQ and SP follow major behaviour-change communications in 2008–2009 by the Ministry of Health. However, it is the first time where information is also available from the private sector to assess the exact decrease in national-level product supply.

Analysis of the data specifically for 2011 provides an additional insight into alternative dynamics with, for example, a shift to artemether and SP as alternative treatment options when faced with a shortfall in the first-line recommendation (AL), despite the concern about relatively low efficacy levels for SP. There are relatively few treatment options for malaria, given the high levels of resistance to older drugs, and the painstaking nature of new drug development in the field. Market data such as these provide useful insights into the possible exposure to less efficient medicines. They are also useful to guide rational use in other therapeutic areas where a wider array of treatment options exists, or there may be cost-effectiveness or other drivers influencing drug utilization decisions. Of particular relevance in for malaria treatment is the question ‘*does antibiotic use increase as more fevers which would previously have been presumptively treated as malaria are confirmed by diagnostic testing not to be malaria?*’. This dynamic between therapeutic areas is an interesting area to explore further and can be done with this system which collects information on all pharmaceutical products, irrespective of therapeutic area or indication.

Such information can also be particularly useful where there may be new developments in disease burden, disease control, or indeed new treatment options available. The ability to track the decrease in use of ineffective medicines such as CQ and SP is of significant importance to policy makers who work with clinicians and who provide behaviour-change messaging to the general public to ensure that there is greater demand for, and prescription/provision of efficacious treatment options or efficacious products with better acceptability profiles. Several new treatment options were indeed made available in for malaria treatment around this time, most notably the availability of a dispersible tablet form (same molecule and strength) of the recommended first-line treatment for children, i.e. those most affected by malaria. Using this system, public health practitioners can better monitor the uptake of such products against alternatives and against disease burden profiles. The benefits of such systems increase further where the market is more diverse, with a larger private sector for example.

In addition to measure of drug utilization and access, market data of this kind can also be used to monitor other policies: the example provided above regarding imports of oral artemisinin monotherapy are of significant importance in the malaria field in relation to efforts to reduce the risk of drug resistance. In covering all products and providing the opportunity for both relatively real-time data as well as multi-year trend analysis, a system of this kind offers a cost-effective solution for analyses on a range of different issues the more the system is used.

Finally, this example highlights opportunities around wider supply side dynamics. As indicated, there is a high concentration among a few manufacturers and importers, with 3–4 manufacturers and up to 9 importers influencing the market overall. However, it is interesting to note that many manufacturers maintain their marketing authorization and remain present in the market, as shown by the large number of products registered with extremely low market share and low sales figures. This information is valuable to public health practitioners and policy makers seeking to optimize access to efficacious, affordable essential medicines. It is also important for investors involved in medicines R&D, production or supply. Such data benefit the regulatory authority which can direct its limited resources most effectively to carry out targeted post-marketing surveillance.

Greater availability of market data, even at an aggregate level and recognizing commercial sensitivities, could provide a great benefit in supporting investment decisions, reducing risk and ensuring that commercial investment decisions are balanced with public health interests such as sufficient and sustainable supply of affordable, quality essential medicines. It should also be noted that the data contain commercially sensitive information: appropriate measures must, therefore, be put in place to balance public health and commercial interests.

### Study limitations

Study limitations include a small number of coding errors in the system which have been identified through detailed data analysis. These will be corrected in future data entry. The absence of information on manufacturer at MSL limits the ease of certain analyses. However, as public bodies, MSL and MoH maintain this information through other means and the data could be cross-referenced if required. Finally, certain components of the data are proprietary to IMS Health (IQVIA) and may only be accessed with permission. This did not negatively influence this study, and similar systems could be established in other countries without such limitations. It should be noted that the dataset comprises all pharmaceuticals: thus, similar analyses could be carried out for other therapeutic areas or drug classes.

Drug utilization studies often make use of the defined daily dose (DDD) to evaluate the supply of medicines as compared to usage. The DDD is defined as ‘*the assumed average maintenance dose per day for a drug used for its main indication in adults’* [20]. It is used to standardize drug usage between drugs and between healthcare environments. The DDD does not relate to actual prescriptions of a medicine, but can be used to compare drug usage relative to disease burden and estimated need. However, malaria treatments are a short course (3 days), with a large portion of the disease burden falling on children under 5 years of age. Many of the newer products are packaged in single treatment units—this is the case for most artemisinin-based combinations, such as AL. Thus, one weight-specific pack of AL represents the appropriate complete treatment course for a patient. As such, DDDs were not calculated for this study, as the count of AL packs per weight band should provide a direct indicator of the supply requirement relative to the estimated disease burden.

## Conclusion

The challenge of gaining a better understanding of total national pharmaceutical markets in middle and low-income countries has been a neglected area of investment for many years and is a missing piece to of the access to medicines puzzle. The absence of systematic data of this kind presents a constraint for researchers and policy makers alike. The importance of full national level total market data is now recognized by key stakeholders in a number of important policy documents, focused on public health, access to medicines, resource allocations and local manufacturing decisions.

This study shows how data can be systematically gathered at national level to monitor and understand more detailed trends in the market. This collaboration has been the first of its kind in Africa to produce a systematic, rolling national market monitoring system with complete national pharmaceutical data at product level, showing market size by value and volume with trend data over multiple years.

As such, the data are invaluable for supporting national analyses, for decision making and for monitoring changes in medicines policy or building the case new policies. They allow the Regulatory Authority and Ministry of Health to monitor and react more quickly to changes which might affect medicines quality, affordability, policy or security of supply and creating a positive policy dialogue among stakeholders.

The analysis of the anti-malarials market highlights the interplay between the public and private sector. The system emphasizes how access to complete and comparative data for both sectors allows analysis of the complementarity of different sectors, as well as highlighting key areas which must be addressed to ensure a middle or low-income country can optimize access to high quality affordable medicines for the whole population, through different delivery channels.

The data provide an evidence-base to support the Regulatory Authority in its responsibility for regulating and monitoring the pharmaceutical market in the country. They allow for more targeted investments, whether financial, resource allocation or follow up on public health goals. They allow the Ministry of Health to ensure that the best medicines are indeed available in the quantities needed in the preferred sectors based on treatment-seeking preferences. Beyond the immediate national priorities, and given the limited commercial value of the market for anti-malarials in high-income countries, such data also contribute to advocacy for the resources required to research, develop, produce and distribute medicines for neglected diseases in middle and low-income countries.

The use of such data should be rapidly expanded, while respecting constraints required to protect commercial confidentiality. This could include increased use of the data in Zambia to other therapeutic areas and application to current policy questions. Such information could, in particular, be applied when considering the interplay across therapeutic options, for example impact of changes in malaria treatment on the use of antibiotics among paediatric populations. Similar data systems could also be rolled out in other countries, allowing comparative analyses across a number of key populations and policy areas. Finally, data can be used at global level to help guide investments in access to medicines and advance drug utilization studies in low- and middle-income countries.

## Abbreviations

ACT: artemisinin-based combination therapy
AL: artemether–lumefantrine
AS: artesunate
AS-SP: artesunate sulfadoxine–pyrimethamine
CQ: chloroquine
DHA–PQP: dihydroartemisinin–piperaquine
DHS: demographic and health survey
EML: essential medicines list
GF: the global fund to fight HIV/AIDS, TB and malaria
IM: intramuscular
Inj.: injectable
IV: intravenous
MDG: millennium development goals
MIS: malaria indicator survey
MoH: Ministry of Health
MSL: Medical Stores Limited (the Central Medical Stores of Zambia)
MMV: Medicines for Malaria Venture
PMI: President’s Malaria Initiative
PQ: WHO Pre-Qualification
PSI: population services international
RDT: rapid diagnostic test (for malaria)
SDG: sustainable development goal
SP: sulfadoxine–pyrimethamine
SRA: stringent regulatory authority
STG: standard treatment guidelines
USD: US dollar
WHO: World Health Organization
ZAMRA: Zambia Medicines Regulatory Agency
